# Good Choice of Electrode Material as the Key to Creating Electrochemical Sensors—Characteristics of Carbon Materials and Transparent Conductive Oxides (TCO)

**DOI:** 10.3390/ma14164743

**Published:** 2021-08-22

**Authors:** Anna Cirocka, Dorota Zarzeczańska, Anna Wcisło

**Affiliations:** Department of Analytical Chemistry, Faculty of Chemistry, University of Gdansk, ul. Wita Stwosza 63, 80-308 Gdansk, Poland; dorota.zarzeczanska@ug.edu.pl

**Keywords:** contact angle, conductive materials, electrochemical measurements, FTO electrodes, carbon electrodes

## Abstract

The search for new electrode materials has become one of the goals of modern electrochemistry. Obtaining electrodes with optimal properties gives a product with a wide application potential, both in analytics and various industries. The aim of this study was to select, from among the presented electrode materials (carbon and oxide), the one whose parameters will be optimal in the context of using them to create sensors. Electrochemical impedance spectroscopy and cyclic voltammetry techniques were used to determine the electrochemical properties of the materials. On the other hand, properties such as hydrophilicity/hydrophobicity and their topological structure were determined using contact angle measurements and confocal microscopy, respectively. Based on the research carried out on a wide group of electrode materials, it was found that transparent conductive oxides of the FTO (fluorine doped tin oxide) type exhibit optimal electrochemical parameters and offer great modification possibilities. These electrodes are characterized by a wide range of work and high chemical stability. In addition, the presence of a transparent oxide layer allows for the preservation of valuable optoelectronic properties. An important feature is also the high sensitivity of these electrodes compared to other tested materials. The combination of these properties made FTO electrodes selected for further research.

## 1. Introduction

An important aspect determining the application potential of new electrodes is to know their electrochemical and surface properties. The new electrode material is expected to have high electrical conductivity, fast electron transfer for a wide range of redox systems, as well as structural and electrochemical stability over a wide range of potentials. An additional advantage of an ideal electrode is its simplicity and low production cost. Meeting these expectations is a guarantee of using a given electrode material as a modification platform in further electroanalytical tests.

The need to quantify various chemical compounds in environmental, medical, or industrial analysis motivates scientists to search for new electrode materials with well-defined electrochemical properties. Embedding molecules with specific properties on the surface of electrode materials allows them to be used as molecular recognition systems in many analytical aspects. As the base material, semiconductors (silicon) or dielectrics (glass, ceramics) are usually used, which, as a result of the formation of conductive or semiconductor structures on their surface, obtain interesting properties from the point of view of electronic technology and physicochemical issues. Most often, we apply chemically defined layers of inorganic oxides and carbon materials with various electrical properties to the surface of the starting material.

The most popular electrode material in electroanalytical measurements is glassy carbon (GC) [1]. It is a carbon material with a very complex structure, consisting of intertwined graphite fibers that do not show long-range order. The presence of the sp^2^ hybridized carbon atom in the structure is associated with the presence of various types of oxygen groups located at the end of the fibers, which may interact with the analyte. Unlike other structures, it is characterized by a glass fracture and the possibility of polishing its surface to a mirror effect. This non-graphite carbon material combines the properties of glass and ceramics with those of graphite. It is impermeable to gases and liquids, has a compact isotropic microstructure, is characterized by high hardness, and has high thermal and chemical resistance, which is superior to other forms of carbon structure used as electrodes. Vitreous carbon is susceptible to machining, which allows for the production of various electrode structures, in the form of rods, plates, or disc electrodes [2,3]. Glassy carbon electrodes are characterized by high electrochemical stability over a wide range of potentials, while showing low electrical resistance. These properties make the glassy carbon electrode an ideal substrate for many electroanalytical tests. Despite the passage of time, the GC electrode is still one of the most used electrode materials in electrochemistry. This is proven by scientific articles describing new application possibilities of this material [4,5,6].

The inclusion of a boron dopant in the diamond crystal structure of sp^3^ hybridization creates very interesting p-type semiconductor materials known as boron doped diamond (BDD) electrodes. BDD electrodes show excellent electrochemical properties [7,8,9]. Compared to other materials used in electroanalytical measurements (Au, Pt, or GC), they are characterized by a wide range of potentials in aqueous and non-aqueous environments, low capacitive current, and microstructural stability at extreme cathode and anodic potentials. High stability, combined with biocompatibility, chemical inertness, and mechanical resistance to contamination make it an interesting material for the construction of electrochemical sensors [10,11,12,13,14,15,16]. Moreover, the BDD electrodes, due to the aliphatic nature of the surface composed of sp^3^ hybridized carbon atoms, show poor adsorption properties. Nevertheless, the lack of chemically reactive functional groups in the structure of this material precludes direct attachment of organic compounds to its surface [17]. The major part of research on BDD electrodes concerns surface modification, enabling covalent coupling of organic modifiers or metal nanoparticles [18,19]. BDD electrodes are used in classical electroanalytical measurements concerning the determination of redoxactive compounds, but also in the construction of biosensors [20,21,22,23].

Thin carbon films made of boron doped nanocrystalline diamond (B-NCD) is another interesting material. It is usually synthesized on silicon substrates, but it can also be successfully obtained on the surface of an amorphous dielectric—quartz glass [24,25]. It has optical parameters similar to quartz and, at the same time, due to the electrical conductivity of the diamond layer, it enables electrochemical measurements [26,27]. The undoubted advantage of optically transparent diamond electrodes is the transmission in a wide range of optical radiation, from ultraviolet to far infrared [28]. However, an important aspect is also the high refractive index of the B-NCD film, which allows a clear optical contrast between the diamond electrode and the glass substrate to be obtained (n_diamCVD_ = 2.4: n_quarc_ = 1.45). Boron-doped diamond films are commonly used as an electrode material for the construction of biosensors by functionalization of the B-NCD surface with DNA [29,30]. Moreover, these nanocrystalline structures have found application as optically transparent electrodes (OTE) for spectroelectrochemical measurements [31].

Intensive research on carbon nanotubes contributed to the discovery of the so-called carbon nanowalls (CNWs), which are a system of graphite walls set vertically to the substrate [32]. Carbon nanowalls are variously branched networks with a morphological structure resembling a labyrinth [33,34,35,36]. The basic properties of carbon nanowalls, which are of fundamental importance for their potential applications, are primarily the interesting structure of the material, i.e., sharp edges or a high surface-to-volume ratio, which makes it an ideal functional support for synthesizing a new composite material with a large area. CNWs have also been used in energy storage, as electrodes for fuel cells, catalyst carriers, lithium-ion batteries or field emission devices [37,38,39,40,41,42]. In addition, the unique properties of CNWs make these electrodes an interesting starting material for the creation of electrochemical sensors for environmental, medical, and industrial analysis [21,43,44,45].

One of the most frequently and intensively studied oxide compositions, due to high conductivity and transparency compared to SnO_2_ or ZnO, is indium tin oxide (ITO) (In_2_O_3_: Sn). Nevertheless, due to the low availability of indium, and thus the increasing costs associated with the production of this electrode, new materials were sought with comparable optoelectronic properties. The FTO (fluorine doped tin oxide) electrode turned out to be an alternative to the ITO electrode. It is a glass covered with a thin layer of conductive inorganic material, fluorine doped tin oxide (SnO_2_: F) [46,47].

FTO electrodes were found to be a very promising material due to their greater weather stability and resistance to high temperatures, compared to ITO electrodes. Moreover, this material is chemically inert, mechanically resistant, and has a high resistance to physical abrasion [48,49]. The key feature of these electrodes is the combination of optical and electrochemical properties. Both FTO and ITO electrodes find practical application in a wide range of devices; among others, they are used to create transparent conductive coatings, in touch panels, flat screens, aircraft cockpit windows, and plasma monitors. Thin oxide layers are also used in the production of organic light emitting diodes (OLED) and in solar cells [50,51]. Moreover, thanks to their unique properties, these materials are a good substrate for modifying their surface [48,52,53,54,55,56]. They are also used in classical electroanalytical measurements as working electrodes in the determination of a wide range of electroactive compounds [57].

The aim of this study was to select, from among the presented electrode materials (carbon and oxide), the one whose parameters will be optimal in the context of using them to create sensors. Based on the research carried out on a wide group of electrode materials, it was found that transparent conductive oxides of the FTO type exhibit optimal electrochemical parameters and offer great modification possibilities. These electrodes are characterized by a wide potential range and high chemical stability. In addition, the presence of a transparent oxide layer allows for the preservation of valuable optoelectronic properties. An important feature is also the high sensitivity of these electrodes compared to other tested materials. The combination of these properties resulted in them being selected for further research. Electrochemical impedance spectroscopy and cyclic voltammetry techniques were used to determine the electrochemical properties of the materials. Properties such as hydrophilicity/hydrophobicity and their topological structure were determined using contact angle measurements and confocal microscopy, respectively.

## 2. Materials and Methods

### 2.1. Electrochemical Measurements

Cyclic voltammetry measurements were carried out in an aqueous solution of Na_2_SO_4_ (0.5 M) containing a reference redox system. Appropriate redox systems, i.e., potassium ferri/ferrocyanide ((Fe(CN)_6_)^3−4−^), hydroquinone/quinone (H_2_Q/Q) at 5 mM concentration were selected for the study. The prepared solutions were deoxidized with a stream of inert gas (argon). Electrochemical measurements were made in a standard three-electrode system consisting of a working electrode (GC, Si/CNW, Si/B-NCD, glass/B-NCD, ITO, and FTO), a reference electrode (silver wire coated with a layer of silver chloride (Ag/AgCl), immersed in a saturated solution of potassium chloride (0.1 M KCl)) and anti-electrodes (platinum). The surface of the working electrode exposed to the electrolyte was about 0.50 cm^2^ or 0.13 cm^2^. Measurements were recorded at appropriate potential scan rates, i.e., 10, 50, and 100 mV/s. Electrochemical experiments were carried out using the Autolab PGSTAT30 potentiostat/galvanostat (Metrohm Autolab B.V., Utrecht, The Netherlands) and Nova software (1.11, 2005–2013, Metrohm Autolab B.V, Utrecht, The Netherlands).

### 2.2. Contact Angle Measurements

The wettability of the electrode materials surface was determined by measuring the contact angle at room temperature using standard liquids based on the static method of a sessile drop. Water, formamide, diiodomethane and glycerol were chosen as measuring liquids with known surface tension. The contact angle values given are mean values measured at various positions on the electrode surface. Drop shape analysis (with a volume of 2 µL or 4 µL) was carried out using the circle method and the Young-Laplace method [58,59,60]. Measurements were made using a KRÜSS Drop Shape Analyzer—DSA100 (Hamburg, Germany). Free surface energy and its components were determined based on the results of direct contact angle measurements calculated by means of the OWRK method [61,62,63,64,65,66,67].

### 2.3. Electrode Materials

The subjects of the study were electrode materials on silicon or glass substrates. Based on the differences resulting from the structure of the conductive layer, the materials were divided into two groups. The first group were FTO glass electrodes. In this case, the conductive layer was transparent fluorine doped tin oxide (SnO_2_/F). These electrodes were purchased from Sigma Aldrich (St. Louis, MO, USA). For FTO electrodes, the layer resistance was ~7 and ~13 Ω/sq, respectively, with a transparency of 80–82% and 82–84.5%. In addition to transparent conductive coatings, the subject of the study were electrodes made of conductive carbon layers deposited on silicon or glass substrates. This group of electrodes includes carbon nanowalls (CNW) and nanocrystalline boron doped diamond (B-NCD). The BDD and TCO electrodes required preliminary surface preparation. These materials were treated to obtain a hydrogenated BDD surface and an oxidized surface of the TCO electrodes [12,18,61,68]. For comparison purposes, popular carbon electrodes, boron doped diamond (BDD), and glassy carbon (GC) disk electrode were selected. As part of the study of the same electrode materials, they were differentiated in terms of chemical composition. Introducing different boron content into the structure of carbon layers, they were given new electrochemical properties. Therefore, the B-NCD electrodes name includes the ratio of boron to carbon atoms (B)/(C) determining the degree of doping. For example, an electrode described as Si/B-NCD-2k has a (B)/(C) ratio of 2000 ppm. The synthesis of carbon layers (BDD, CNW, and B-NCD) on the silicon/quartz glass surface, along with the selection of deposition parameters, was carried out in accordance with the procedures described in earlier works [18,25,31,33,69] by the team of Prof. Robert Bogdanowicz from the Department of Metrology and Optoelectronics from the Faculty of Electronics, Telecommunications and Informatics of the Gdańsk University of Technology.

## 3. Results and Discussion

Searching for new materials that could form the platform for future sensors, a number of conductive materials that differed in both chemical and surface structure were investigated. Among the tested group of electrodes were three types of carbon electrodes (Si/CNW, Si/B-NCD, and glass/B-NCD) and one TCO electrode (FTO). Due to the fact that the subjects of the study were electrodes with different chemical composition and surface structure, differences in properties were expected. The operation of the sensor based on the modified electrode is represented by an electrochemical signal (or a change in the impedance spectrum). Therefore, these materials are tested using standard redox systems to assess their properties and choose the one with the best parameters. The model system (Fe(CN)_6_)^3−/4−^ is one of the most frequently chosen analytes to assess the electrocatalytic performance of electrode materials. As an outer sphere system, sensitive to changes in the construction of the electrode layer, it is a perfect model to characterize a wide range of materials. Nevertheless, the conducted research showed that on carbon materials, especially those characterized by different hybridization of the carbon atom, this system behaves completely differently.

Due to the fact that specific interactions play an important role in the reactions of the outer sphere, we decided to use the measurements of wettability and free surface energy parameters to assess these phenomena.

### 3.1. Electrochemical Properties

**Figure 1:**
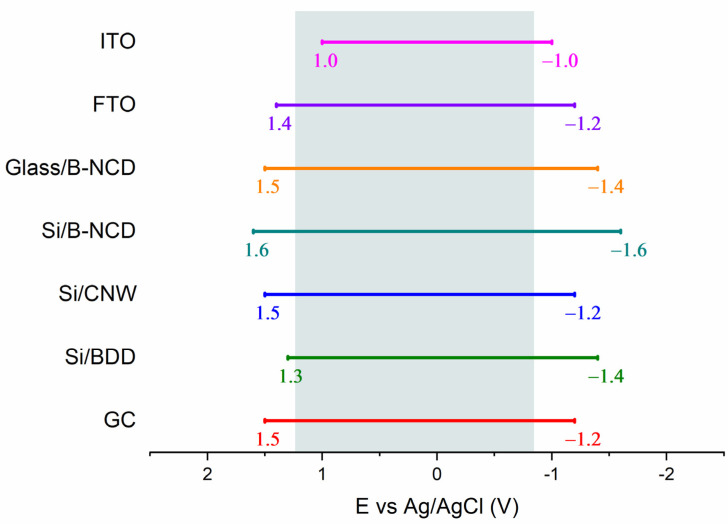
Schematic representation of the range of electrochemical stability of the tested group of electrodes in 0.5 M Na_2_SO_4_ together with the designated area in which water electrolysis takes place (gray field).

Determining the characteristics of electrode materials usually begins by determining the potential range in which the work of the electrode is possible. The wider it is, the larger the group of analytes that differ significantly in oxidation and reduction potential which can be studied. Each of the tested electrodes is characterized by a so-called wide “electrochemical window” (Figure 1).

The electrochemical stability range of classic GC and Si/BDD electrodes is 2.7 V, which is comparable with other carbon materials under investigation. The Si/B-NCD electrode deserves special mention, as it is the only one with a potential range above 3 V. The wider stability range results from differences in the structural properties of diamond electrodes. It matters whether they are microcrystalline (BDD) or nanocrystalline boron doped diamonds (B-NCD). According to the available literature, ultra-nanocrystalline (UNCD) diamonds with crystals smaller than B-NCD are even more stable [70]. Undoubtedly, despite the wide range of Si/B-NCD electrode potentials, the disadvantage is the presence in the anode range of a peak at a potential of 1.2 V. This peak is associated with a carbon oxidation reaction with sp^2^ hybridization occurring at the grain boundary, where electrochemically active carbon–oxygen forms can be formed [31,70]. Comparing B-NCD electrodes with each other, it seems that the type of substrate affects the range of the electrode. However, these materials could differ in the rate of carbon layer growth during the deposition process, which in turn resulted in electrodes of different thicknesses. The growth time was 1 h for silicon substrates and 3 h for glass substrate. In the first case nanocrystalline layers less than 100 nm thick were obtained, in the second case about 250 nm thick [25,31]. It should be noted that this is a factor that significantly affects the range of potentials. Therefore, a direct comparison of electrochemical stability exhibited by a very thin and thick layer of boron-doped diamond is not reliable [70,71]. The ITO electrode (2 V) has the narrowest potential range in 0.5 M Na_2_SO_4_. The narrow operating range of the electrode from the group of transparent conductive oxides is related not only to the decomposition of the supporting electrolyte, but also to the gradual disappearance of the optical and electrical properties of the oxide layer in far cathode ranges. Descriptions in the literature studies indicate that during anodic polarization the substrate undergoes slow degradation by oxidation of the ITO oxide layer. The nature of the ions present in the supporting electrolyte and the exposure time of the electrode in the solution strongly affects the rate of electro-dissolution in the anodic and cathodic range [72,73]. On the other hand, the FTO electrode is characterized by electrochemical stability similar to that of carbon materials. The potential range is wider by 0.6 V compared to the ITO electrode, which proves a better chemical stability of this material in 0.5 M Na_2_SO_4_. The reactions related to the electrolysis of the supporting electrolyte at the FTO electrode are similar to those of ITO, but the signal intensity is much lower. It is worth noting that the literature does not mention any information about the degradation of the conductive layer in the anode range, as is the case with the indium-tin electrode, which is an additional advantage of this electrode [73]. The wide operating range of the FTO electrode while maintaining valuable optoelectronic properties makes this material the most suitable material for further electroanalytical research.

**Figure 2:**
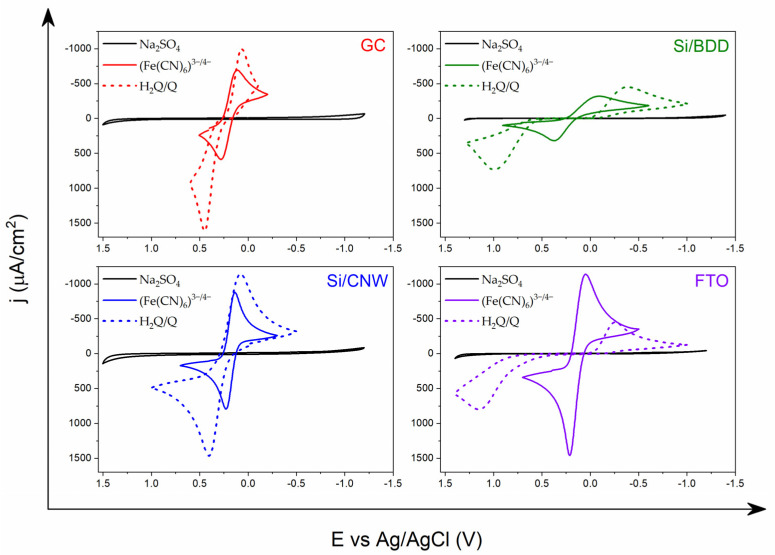
Comparison of cyclic voltammograms of the reference redox systems (Fe(CN)_6_)^3−/4−^ and H_2_Q/Q in an aqueous solution of Na_2_SO_4_ (0.5 M) registered on the following electrodes: glassy carbon (GC), boron doped diamond on silicone (Si/BDD), carbon nanowalls on silicone (Si/CNW) and fluorine doped tin oxide (FTO). Scan rate: 0.1 Vs^−1^.

In sensory applications, an important feature is the sensitivity of the system to a given analyte. Therefore, another important feature is the high level of depolarizer signal in relation to the background, which is associated with a low capacitive current that allows us to reduce the level of interference and, consequently, to lower the detection limit of the analyte. The presence of electroactive labeled substances in the solution, (Fe(CN)_6_)^3−/4−^ or H_2_Q/Q (Figure 2), generates the appearance of electrochemical signals on voltammograms. The differences in the intensity of the analytical response illustrate the different behavior of selected redox systems in relation to the tested electrode materials. This is influenced by many factors originating both from the electrode itself and from the redox system, which was analyzed in the presented studies.

The recorded voltammograms for the model (Fe(CN)_6_)^3−/4−^ in Na_2_SO_4_ (Figure 2) confirm that the rate of this redox reaction is strongly influenced by the nature of the surface exposed to the solution. Electrochemical reversibility for this redox process is much better on sp^2^ carbon electrodes than on a classic diamond electrode. The very slow electron transfer kinetics on the Si/BDD electrode is demonstrated by the high value of ΔE, as well as low values of current densities, j_a_ and j_k_ (Table 1). The best reversibility for the redox process (Fe(CN)_6_)^3−/4−^ was observed on the Si/CNW electrode, where the ΔE value is up to five times lower compared to the Si/BDD electrode, and is only 0.092 V. Moreover, with this electrode material the current density is more than twice as high for the anodic and cathode responses as compared to the diamond electrode. For comparison, the separation of the oxidation and reduction peaks on a classic GC electrode is 0.171 V. For the B-NCD-10k electrode on a silicon substrate, the shift of the oxidation peak (Fe(CN)_6_)^3−^ to (Fe(CN)_6_)^4−^ is observed—towards more positive potential values (0.328 V) and the reduction peak towards negative values (0.022 V), which results in increased peak separation of 0.306 V. This type of behavior was observed on the Si/BDD electrode. In turn, the electrochemical parameters of the B-NCD-10k (Table 1) electrode on a glass substrate are oriented at values close to the Si/CNW electrode, as ΔE is 0.126 V, and the current response signals have similar densities. Of all the electrode materials tested, the FTO electrode deserves special mention. The electrochemical parameters recorded for the oxidation and reductions reaction of the (Fe(CN)_6_)^3−/4−^ system are extremely interesting. Despite the similar reversibility to the GC electrode, oscillating around 0.165 V, it shows much higher current signals. This proves that the detection limit of the FTO electrodes is significantly lower for this system compared to other electrodes. The additional benefit of this electrode is that the conductive layer is not a carbon material, but a transparent inorganic oxide (SnO_2_: F).

Figure 2 also shows the electrochemical behavior of the hydroquinone/quinone model system. According to the literature, the redox reaction is not affected by the presence of oxidized groups on the surface of the electrode material, and electron transfer is preceded by the adsorption step of the depolarizer particles [74].

For the hydroquinone/quinone pair, a typical oxidation/reduction reaction of single peaks was observed in Figure 2, which is the electrode response for the transfer of two electrons and protons on a GC substrate. More significant effects were observed in the case of carbon nanowalls. With respect to the classic GC electrode, the electrochemical reversibility for this process on the surface of the Si/CNW electrode increased by 63 mV. Similar properties were observed when modifying the GC electrode with single-walled carbon nanotubes (SWCNT). SWCNT as cylindrical rolled graphene layers contributed to an increase in the reversibility of the redox process due to adsorption of hydroquinone on the walls of carbon nanotubes. This phenomenon was confirmed by narrow, symmetrical oxidation and reduction peaks on the CV curve of the H_2_Q/Q system for a modified GC surface [74]. As carbon nanowalls belong to the same group of carbon materials as nanotubes, similar effects were expected from the H_2_Q/Q system. It is worth noting that the voltammograms obtained for the Si/BDD and FTO electrodes are completely different (Figure 2). The analyzed redox system shows different behavior on a carbon surface rich in carbon atoms with sp^3^ hybridization and on transparent oxide coatings. Oxidation and reduction peaks are characterized by increased separation and lower current density. ΔE takes values up to 1 V with a twice lower current response signal compared to sp^2^ carbon electrodes. This demonstrates the lower affinity of this redox system to the surface of Si/BDD and FTO electrodes, which results in inhibition of the electron transfer process. A significant improvement in electron transfer kinetics on the Si/CNW surface was observed compared to GC or BDD electrodes in the case of redox pair having delocalized π electrons in their structure. This manifests itself in a different behavior of the H_2_Q/Q system relative to (Fe(CN)_6_)^3^^−/4^^−^. This may indicate the interaction of π–π between organic molecules and carbon walls on the surface of the Si/CNW electrode.

**Figure 3:**
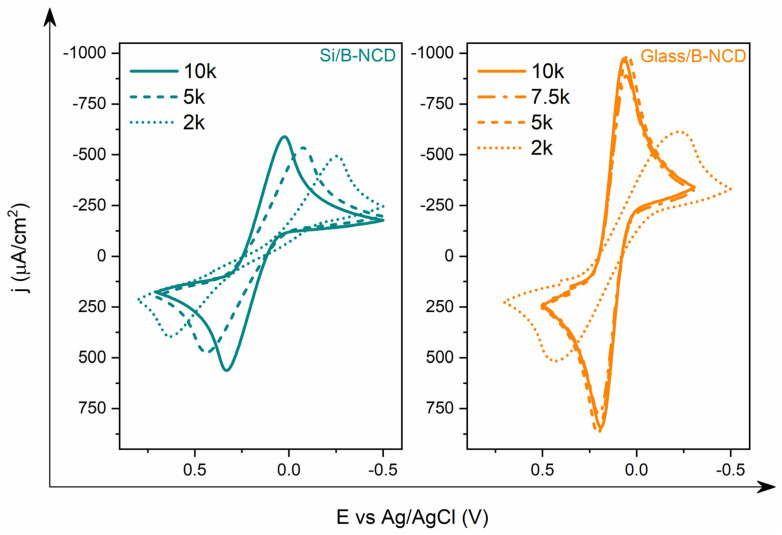
Comparison of cyclic voltammograms of the model redox system (Fe(CN)_6_)^3^^−/4^^−^ (5 mM) in an aqueous solution of Na_2_SO_4_ (0.5 M) registered on B-NCD electrodes on silicon and glass substrate with different (B)/(C) ratios.

As the level of boron doping increases, the electrochemical properties of the electrodes change (Figure 3). On the basis of obtained cyclic voltammograms of B-NCD electrodes, it can be observed that the redox reaction rate increases with an increasing (B)/(C) ratio, which is due to the increased density of electronic states. The highest value of ΔE was recorded for the Glass/B-NCD-10k electrode (0.126 V), which indicates greater efficiency of electron transfer compared to other electrodes, and above all for the Glass/B-NCD-2k electrode (0.657 V) (Table 2). The largest changes in the reversibility of the redox process were noted for Si/B-NCD electrodes. The difference in ΔE between the electrode with the largest doping (10k) and the smallest (2k) is 585 mV. Although it is the same conductive material, it exhibits different behavior relative to the electron transfer process for the (Fe(CN)_6_)^3^^−/4^^−^ system. This is due to the different growth time of the carbon layer, and consequently the thickness of the conductive material, and not the substrate on which the layer was deposited, which was noted when determining the electrochemical stability of these electrodes [25,31].

### 3.2. Wettability

**Figure 4:**
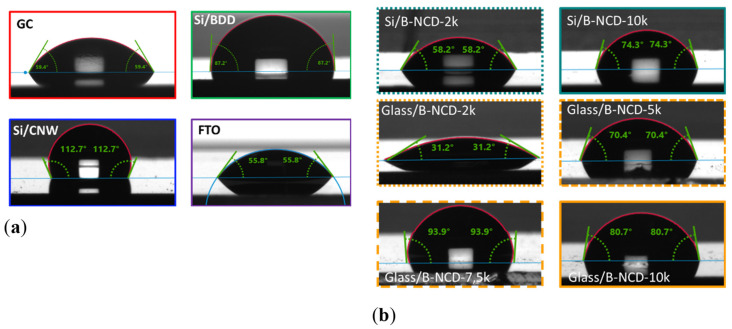
Pictures of contact angle measurement (WCA) of electrode materials (**a**) GC, Si/BDD, Si/CNW, and FTO; (**b**) boron-doped nanodiamonds (B-NCD) on glass and silicon substrates with different (B)/(C) ratios.

The tested electrode materials are characterized by different wettability. The most hydrophobic surface has an Si/CNW electrode (112.79°) (Figure 4a), and a hydrophilic nanodiamond doped with boron on a glass substrate with 2000 ppm doping (31.20°) (Figure 4b). The value of the contact angle for the FTO electrode surface (55.93°) indicates that it is also a material with hydrophilic properties. This is undoubtedly due to their chemical nature, but also to differences in the surface roughness.

The research shows that, regardless of the substrate (glass, silicon) used, electrode materials with a higher degree of doping are characterized by higher hydrophobicity, which is visible at higher contact angle (>70°) (Figure 4b).

### 3.3. Surface Energy

Surface Free Energy (SFE) is the effect of intermolecular interactions at the liquid–solid interface, and allows us to describe these interactions. It can be divided into two components—(1) disperse (γ^D^), attributed to van der Waals interactions and other non-specific interactions, and (2) polar (γ^P^) resulting from dipole–dipole, dipole-induced dipole, hydrogen bonds, and other specific interactions at the liquid–solid interface [75].

**Figure 5:**
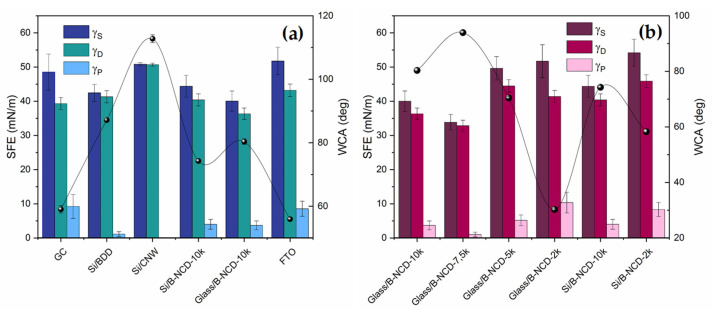
Free surface energy (SFE) graph and contact angles of tested conductive materials: (**a**) GC, Si/BDD, Si/CNW, Si/B-NCD-10k, Glass/B-NCD-10k, and FTO; (**b**) boron doped nanodiamonds (B-NCD) on glass and silicon substrates with different (B)/(C) ratios; γ^S^-SFE, γ^D^: disperse part, γ^P^: polar part, and WCA: water contact angle.

All tested conductive materials have a high disperse component value and a low polar component value, but they differ significantly (Table 3). As the polar component increases, the surface hydrophilicity increases. The largest share of the polar part (approx. 8–9 mN/m) has GC and FTO electrodes, which confirms the contact angle (approx. 55–59°) (Figure 5a). In the case of carbon materials, a greater degree of boron doping results in a reduced proportion of the SFE polar part, and therefore most surface interactions will be dispersive (Figure 5b). In addition, the total free surface energy is lower for materials with a larger (B)/(C) ratio.

**Figure 6:**
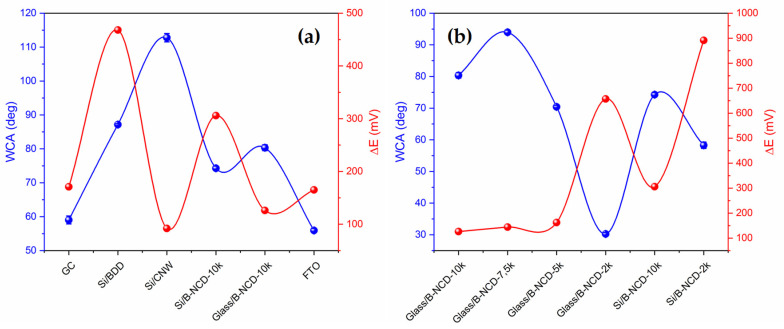
Graph of the water contact angle (WCA) and reversibility of the redox process for (Fe(CN)_6_)^3^^−/4^^−^ couple (ΔE) on the type of electrode: (**a**) GC, Si/BDD, Si/CNW, Si/B- NCD-10k, Glass/B-NCD-10k, and FTO; (**b**) from different levels of doping B-NCD electrodes on a glass and silicon substrate.

**Figure 7:**
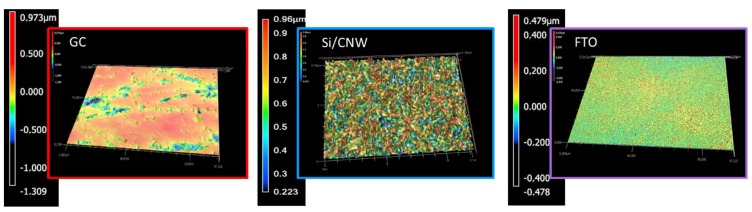
Keyence VK-X1000 confocal laser microscope photos of the surface of electrode materials: GC, Si/CNW, and FTO.

In the case of different materials, the correlation between the contact angle and ΔE is much more complex, which probably results directly from the morphology of the material. The most hydrophobic nature, and at the same time the best reversibility of the redox process, was observed for Si/CNW—these are materials with high roughness (Figure 6a). Such structure of the material may cause the reaction of, in the wetting process, it acting on a drop of water similar to a lotus leaf, by reflecting the drop off the surface of the material [76]. In turn, GC and FTO electrodes are characterized by the highest uniformity of surface structure (Figure 7). Compared to others, both materials are hydrophilic and have good reversibility of the redox process (Figure 6a, Table 1 and Table 3).

In the case of doped B-NCD electrodes, the reversibility of the redox process is opposite to the contact angle—for highly doped electrodes, we observe better reversibility, but weaker wettability. Additionally, for 2k electrodes, weaker reversibility at higher hydrophilicity (Figure 6b).

## 4. Conclusions

In recent years, materials chemistry has been one of the most intensively developing fields of science. It mainly includes the synthesis of new electrode materials or modification of their surface to give them specific properties, increasing their applicability. Among the wide range of electrode materials, there are classic metallic electrodes, such as gold and platinum, as well as electrode materials based on carbon and oxide layers.

Carbon and its various structural forms (graphite, graphene, diamond, nanotubes, and other structural forms with sp^2^ and sp^3^ hybridization) make it possible to use it in a wide range of scientific and technological aspects, due to the great diversity in structure, and thus also in physical and chemical properties.

Despite the availability of many metallic and carbon electrodes on the market, materials with transparent conductive oxides are also very popular. Their important feature is optical transparency, which, combined with high electrical conductivity, allows them to be used in electrochemistry and optoelectronics. The best application properties are demonstrated by electrodes based on zinc, indium, and tin oxide.

The subjects of the presented research were electrode materials on silicon or glass substrates. Based on the differences resulting from the structure of the conductive layer, the materials were divided into two groups. The first group were glass electrodes of the FTO and ITO types. In this case, the conductive layer consisted of transparent inorganic oxides, tin oxide with an admixture of fluorine, and indium-tin oxide. In addition to transparent conductive coatings, the subject of the research were electrodes made of conductive carbon layers deposited on silicon or glass substrates. This group of electrodes includes, e.g., carbon nanowalls (CNW) (being a system of graphite walls set vertically to the substrate) and nanocrystalline boron-doped diamond (B-NCD). For comparison purposes, the popular carbon electrodes, boron doped diamond (BDD) and glassy carbon disc electrode (GC), were selected.

In order to assess how the new materials compare with other commercially available electrodes, their characteristics were made. On the basis of the voltametric measurements recorded in the supporting electrolyte, we determined the potential limits reflecting the operating range of each electrode. Another important aspect that determines the application potential of the electrodes is the high level of the signal of the electroactive substance in relation to the capacitive current of the electrode. To assess the electrochemical reactivity of the analyzed electrode materials, we chose two redox systems: potassium hexacyanoferrate (III)/(II) and the hydroquinone/quinone system.

The conducted research allowed us to choose, from a wide range of tested electrode materials, that with optimal parameters. The FTO electrode is characterized by its distinctive features—electrochemical stability similar to carbon materials and higher compared to the ITO electrode. Moreover, the FTO electrode is characterized by a similar reversibility of the redox process of the model potassium ferrocyanide (III)/(II) system to the GC electrode (165 mV). It shows significantly higher current response signals. This proves a higher level of detection of this electrode for this system compared to other electrodes.

In addition, when examining the wettability, the most hydrophobic surface has the electrode of the carbon nanowalls type (113°), and the hydrophilic FTO electrode (56°). This is undoubtedly due to their chemical nature, but also to differences due to surface roughness. The FTO electrode has the smoothest surface.

The wide operating range of the FTO electrode while maintaining the valuable optoelectronic properties, as well as the high sensitivity of these electrodes compared to other materials tested, meant that we chose the FTO electrode for further electroanalytical tests. In the following works, we will present in detail the modification process of the FTO electrode, the influence of various conditions on the obtained layers, as well as the potential application of the obtained devices.

## Figures and Tables

**Table 1:** Electrochemical parameters of the reference redox systems (Fe(CN)_6_)^3−/4−^, and H_2_Q/Q in an aqueous solution of Na_2_SO_4_ (0.5 M) registered on the following electrodes: GC, Si/BDD, Si/CNW, and FTO.

Electrode	Redox System	E_a_ (V)	E_k_ (V)	ΔE (V)	j_a_ (µA/cm^2^)	j_k_ (µA/cm^2^)
GC	(Fe(CN)_6_)^3−/4−^	0.283	0.112	0.171	585.5	−692.9
	H_2_Q/Q	0.450	0.063	0.387	1617.4	−993.7
Si/BDD	(Fe(CN)_6_)^3−/4−^	0.376	−0.092	0.468	318.7	−319.3
	H_2_Q/Q	0.990	−0.387	1.377	730.2	−450.7
Si/CNW	(Fe(CN)_6_)^3−/4−^	0.229	0.137	0.092	794.0	−877.1
	H_2_Q/Q	0.403	0.079	0.324	1465.0	−1143.9
FTO	(Fe(CN)_6_)^3−/4−^	0.214	0.049	0.165	1460.2	−1139.6
	H_2_Q/Q	1.151	−0.260	1.411	798.9	−451.9

ΔE = E_a_ − E_k_.

**Table 2:** Electrochemical parameters of the reference redox systems (Fe(CN)_6_)^3^^−/4^^−^ in an aqueous solution of Na_2_SO_4_ (0.5 M) registered on B-NCD electrodes on silicon and glass substrate with different (B)/(C) ratios.

Electrode	(B)/(C)	E_a_ (V)	E_k_ (V)	ΔE (V)	j_a_ (µA/cm^2^)	j_k_ (µA/cm^2^)
Si/B-NCD	10k	0.328	0.022	0.306	562.0	−588.9
	5k	0.436	−0.077	0.513	473.1	−533.4
	2k	0.634	−0.257	0.891	395.8	−493.3
Glass/B-NCD	10k	0.188	0.062	0.126	844.9	−972.8
	7.5k	0.197	0.053	0.144	779.8	−889.1
	5k	0.206	0.044	0.162	875.0	−991.3
	2k	0.436	−0.221	0.657	515.9	−612.8

ΔE = E_a_ − E_k_.

**Table 3:** Contact angle parameters and surface free energy for selected conductive materials.

Measured Value	Electrode	GC	Si/BDD	Si/CNW	FTO
Contact angle Ɵ (°)	Water	59.03 (±1.17)	87.13 (±0.30)	112.79 (±1.23)	55.93 (±0.59)
Surface Free Energy (mN/m)	γ_S_	48.54 (±5.26)	42.46 (±2.49)	50.82 (±0.46)	51.77 (±4.02)
	γ_D_	39.32 (±1.76)	41.32 (±1.77)	50.72 (±0.44)	43.22 (±1.82)
	γ_P_	9.22 (±3.50)	1.14 (±0.72)	0.11 (±0.02)	8.55 (±2.20)

## Data Availability

All the data is available within the manuscript.
